# The Scientific Journal: Authorship and the Politics of Knowledge in the Nineteenth Century

**DOI:** 10.5195/jmla.2022.1406

**Published:** 2022-04-01

**Authors:** Melissa Grafe

**Affiliations:** 1 melissa.grafe@yale.edu, Head of the Medical Historical Library, Harvey Cushing/John Hay Whitney Medical Library, Yale University, New Haven, CT

In the medical library field, the medical and scientific journal article holds a privileged place. Considered the space for unveiling cutting-edge and recent discoveries, reviewing various topics and themes, and situating research in the larger medical and scientific world, journals and journal articles provide legitimacy to the work of researchers and faculty throughout the world. The business of medical libraries supports this scholarly apparatus through journal and database subscriptions, loans, education programs, and staff dedicated to helping researchers in various parts of the research and publication process. In the grind of this cycle, there is often little time to reflect on how and why the scientific journal and article rose to this privileged place and the larger historic roots behind this enterprise. Alex Csiszar's *The Scientific Journal: Authorship and the Politics of Knowledge in the Nineteenth Century* is a deep exploration into Western European scientific cultures and debates surrounding the presentation and privileging of knowledge in nineteenth-century journals. Throughout this volume, Csiszar considers the place of trust, expertise, accountability, public knowledge, and politics, all of which are interwoven into the development of scientific knowledge in the nineteenth century and today.

Csiszar's study centers on episodes of debate and change related to scientific knowledge that occurred between scientific communities, particularly the Royal Society in London and the Royal Academy of Sciences in Paris, and periodical presses in nineteenth-century France and Britain, although he periodically references other European countries. Recent scholarship has delved into the circulation of scientific knowledge, the economic aspects of publishing, and the popularization of science, which are not the main thrusts of Csiszar's work, as he acknowledges in the introduction. Instead, Csiszar weaves together the history of scientific knowledge production and authorship with book history, situating his work in fields dominated by Steven Shapin and Bruno Latour, and works on Victorian science and book readership like James Secord's *Vision of Science* [1]. In some sense, Csiszar's text is reminiscent of books like Anthony Grafton's *The Footnote: A Curious History* [2], which places the use of the footnote into a historical context.

At the heart of Csiszar's work are two questions, asking why the format of the published scientific paper became the main vehicle carrying what he calls the “epistemic weight” (p. 2) of science instead of a book, for example, and how public trust and legitimacy became tied to scientific literature. Not surprisingly, the answer is fluid, and his questions have deep relevance for understanding scientific literature and publication today, which he clearly acknowledges in the introduction and conclusion. Csiszar also questions why the public is important for legitimating scientific knowledge, especially in light of the rise of scientific experts. He explores many other formats that communicate science, such as newspapers, pamphlets, abstracts, catalogs, and letters, to help drive home how remarkable it is that the scientific journal became the seat of scientific legitimacy and authority. Different chapters discuss how the Royal Society and the Royal Academy of Sciences interacted with the larger periodical community, writers, editors, and broader public(s); identified and granted authorship; and developed a referee system (or not). Csiszar also considers what role revolutionary politics in France and England played in the spread or censorship of scientific knowledge, what role the market played in scientific discovery, and how scientists and groups beyond the Society and Academy contributed to debates prioritizing authorship, among many other themes.

This text is certainly intended for academic audiences, although anyone interested in the history of the development of scientific literature can dive into the volume. Csiszar often presents the main questions and thrusts of his argument in multiple ways to ensure the reader gets the point. Periodically, the sheer number of individuals involved in the various scientific societies, journals, publishers, and their critics is hard to keep track of, and having some sense of French and British history of the early to mid-nineteenth centuries is helpful.

Csiszar ends his book with a quick dive into the twentieth and twenty-first centuries, as debates created by new technologies, such as the Internet and preprint repositories, continue highlighting larger issues related to scientific legitimacy and the politics around science in this era. New technologies generate hope, and sometimes fantasy, bound up in the idea that open data and research made available on new platforms will solve some of the deeper problems around scientific production, sometimes ignoring the political and commercial parts of scientific life. Csiszar reminds the reader that genres and formats are fluid, and that scientific literature is still at “a juncture where the cultures of trust that still undergird much of scientific life and the cultures of accountability that dominate much of public life have come together” (p. 288). While COVID-19 has certainly put this assumption to the test, the thousands of articles generated annually by the scientific community continue to center the published scientific paper at the heart of the research enterprise.
